# Usutu Virus RNA in Mosquitoes, Israel, 2014–2015

**DOI:** 10.3201/eid2310.171017

**Published:** 2017-10

**Authors:** Batya Mannasse, Ella Mendelson, Laor Orshan, Orna Mor, Uri Shalom, Tamar Yeger, Yaniv Lustig

**Affiliations:** Chaim Sheba Medical Center, Ramat Gan, Israel (B. Mannasse, E. Mendelson, O. Mor, Y. Lustig);; Medicine, Sackler School of Public Health, Tel-Aviv University, Tel-Aviv, Israel (E. Mendelson);; Ministry of Health, Jerusalem, Israel (L. Orshan);; Ministry of Environmental Protection, Jerusalem (U. Shalom, T. Yeger)

**Keywords:** Usutu virus, USUV, viruses, flavivirus, virus RNA, West Nile virus, WNV, mosquitoes, Israel

## Abstract

We identified Usutu virus (USUV) RNA in 6 pools of mosquitoes trapped in northern Israel during 2014–2015. These Israeli strains were most similar to strains identified in Senegal and Germany, which further elucidates common ancestry and evolutionary dynamics of USUV. Our findings suggest that human infection with USUV might occur in Israel.

Usutu virus (USUV) is a vectorborne flavivirus first isolated in South Africa in 1959 (*1*). The reservoirs for USUV, which are similar to those for West Nile virus (WNV), include numerous species of birds, and it is transmitted primarily by *Culex* spp. mosquitoes (*2*). Circulation of USUV has been reported in several countries in Africa, and 2 human cases of infection were identified in Africa in 1981 and 2004 (*3*). Since 1996, when USUV was first detected outside Africa in a blackbird in Italy, the virus has been identified in mosquitoes and birds in several countries in Europe (*4*). In recent years, there has been increasing evidence for human infection with USUV (*4*), and USUV antibodies and USUV RNA have been detected in blood donors (*5*,*6*).

A study published in 2016 characterized USUV phylogeny on the basis of available USUV strains from Africa and Europe (*7*). This study suggested that multiple introductions of USUV into central Europe from Africa could be confirmed and that Senegal was a possible origin for epizootics in central Europe.

WNV is the only flavivirus detected in Israel. WNV circulates in mosquitoes and birds in this country and was responsible for ≈1,400 cases of human infections during 2000–2012 (*8*). Because Israel is located on a central bird migration path between Africa and Eurasia (*9*), circulation of USUV in this area is also plausible. We used the WNV national mosquito surveillance system in Israel as a source for identification of USUV strains circulating in mosquitoes in Israel during 2014–2015.

## The Study

**Figure 1:**
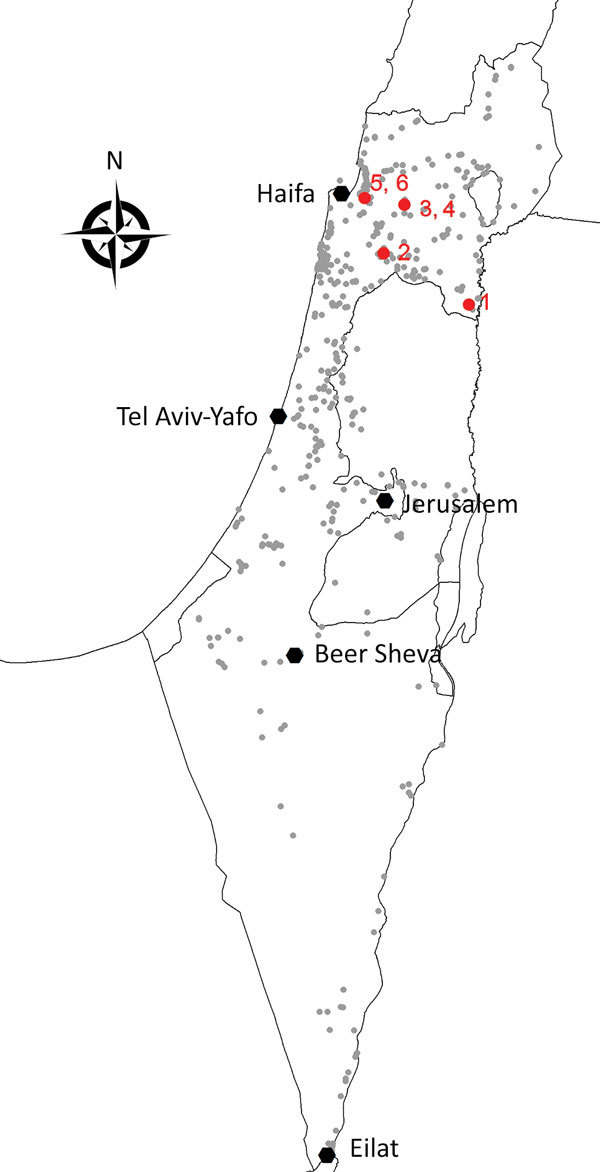
Spatial distribution of collection sites and Usutu virus infectious mosquitoes, Israel 2014–2015. Small gray circles indicate collection sites. Red symbols and numbers indicate sites of Usutu virus–infected mosquitoes.

During 2014–2015, a total of 53,890 mosquitoes were trapped and grouped into 1,471 pools of <50 mosquitoes/pool. We tested RNA extracted from mosquito pools for USUV RNA by using quantitative reverse transcription PCR and specific primer–probe sets for the USUV nonstructural protein 5 (NS5) gene as described (*10*). We detected USUV RNA in 6 pools, 5 from 2015 and 1 from 2014. Although mosquito specimens were collected from trapping sites throughout Israel, all USUV RNA–positive pools were captured in northern Israel (Figure 1) and belonged to 3 mosquito species (Table).

**Table:** Analysis of mosquitoes for Usutu virus, northern Israel, 2014–2015*

Collection site no.	Virus name	Location of collection site	Date of sample collection	Mosquito species	MLE
1	Usutu, 269 m/2015/Israel	Sdeh Eliahu	2015 Jun 30	*Culex perexigus*	0.0047
2	Usutu, 550 m/2015/Israel	Midrach Stream	2015 Sep 8	*Cx. perexigus*	0.0025
3	Usutu, 558 m/2015/Israel	Yeftachel	2015 Sep 8	*Cx. pipiens*	0.0138
4	Usutu, 569 m/2015/Israel	Yeftachel	2015 Sep 8	*Cx. pipiens*	0.0138
5	Usutu, 593 m/2015/Israel	Haifa	2015 Sep 21	*Aedes albopictus*	1
6	Usutu, 610 m/2014/Israel	Kityat Ata	2014 Oct 2	*Cx. pipiens*	0.0041

*Virus-positive mosquitoes were identified by using real-time reverse transcription PCR. MLE for infection rate was calculated by using the EpiTools epidemiologic calculators method (http://epitools.ausvet.com.au/content.php?page = PPVariablePoolSize). MLE, maximum likelihood estimate.

**Figure 2:**
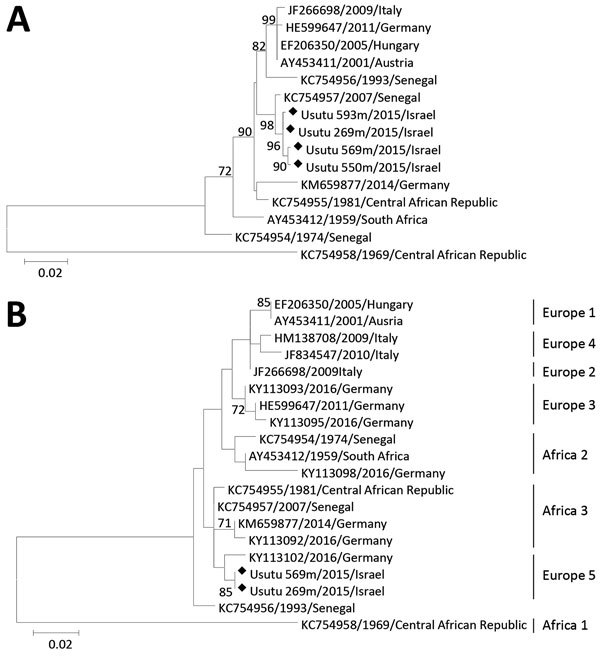
Phylogenetic tree of Usutu virus strains, Israel, 2014–2015. A) Structural protein genes. B) Nonstructural protein 5 genes. Analysis was conducted by using the maximum-likelihood method implemented in MEGA 6.0 software (http://www.megasoftware.net/). Robustness of branching pattern was tested by using 1,000 bootstrap replications. Percentage of successful bootstrap replicates is indicated at nodes (only values >70% are shown). Diamonds indicate virus strains sequenced in this study. Reference strains are indicated by GenBank accession numbers and country and year of isolation. Scale bars indicate nucleotide substitutions per site.

We then performed amplification of an 845-nt sequence containing part of the envelope, membrane, and premembrane genes of USUV as described (*11*). Phylogenetic analysis showed that sequenced USUV strains from Israel clustered with a USUV strain isolated from *Cx. neavei* mosquitoes in 2007 in Senegal (Figure 2, panel A).

We then performed phylogenetic analysis of part of the NS5 gene because a previous study showed that this sequence exhibits a phylogenetic signal similar to the complete genome (*7*), which enables rapid characterization of circulating virus strains. Results (Figure 2, panel B) showed that USUV sequences from Israel closely resemble sequences of a USUV strain isolated from a bird in Germany in 2016, which was recently shown to belong to a putative novel USUV lineage called Europe 5 (*11*).

## Conclusions

Despite its identification in 1959, USUV was not considered a potential public health concern until the early 2000s, when the first large USUV outbreak in birds occurred in Austria (*10*). Since that time, several human cases were identified in Europe and showed a correlation with increasing numbers of birds infected with USUV (*4*). It is possible that, similar to WNV outbreaks in Europe, the United States, and Israel during 1990–2010, which were occasionally preceded by large numbers of WNV infections in birds (*12*), USUV detection in birds and mosquitoes might be the first indication of future USUV outbreaks in humans. In this study, we identified that USUV RNA is present in mosquitoes in Israel, which suggested that USUV infection in humans might also occur in Israel.

USUV RNA has been detected primarily in *Cx. pipiens* and *Cx. perexigus* mosquitoes in Spain (*13*), in *Cx. pipiens* and *Cx. torrentium* mosquitoes in Germany (*14*), and in *Ae. albopictus* and *Cx. pipiens* mosquitoes in Italy (*15*). Our finding of USUV RNA in *Cx. perexigus*, *Cx. pipiens*, and *Ae. albopictus* mosquitoes also suggests that USUV is transmitted by these 3 mosquito species in Israel.

Recently, reconstruction of the evolutionary history and dispersal of USUV has identified 6 distinct lineages of USUV. Nevertheless, Engel et al. concluded that limited numbers of USUV isolates from Africa and lack of data for countries located between Africa and Europe might obscure additional spatial movements (*7*).

Our phylogenetic analysis of the NS5 gene (Table) showed that USUV strains detected in Israel belong to the USUV Europe 5 lineage, mostly resembling a strain isolated in Germany in 2016. However, phylogenetic analysis on the basis of part of USUV structural proteins (Figure 1) indicates that virus strains from Israel are similar to USUV isolated from mosquitoes in Senegal in 2007, which was shown to be part of the Africa 3 lineage (*11*).

Because Israel is located on a bird migration path between Africa and Eurasia, USUV strains isolated in Germany, which are part of the Europe 5 and Africa 3 lineages, might have arrived from Africa through Israel, thus explaining the relative similarity of the strains in Israel to both lineages. Identification of more isolates from the Middle East and in-depth sequence analysis are needed to examine the geographic spread of the virus and further decipher its evolutionary history.

Our results showed that all 6 USUV-positive pools were captured in 3 mosquito species in northern Israel (Figure 1). Because most WNV-positive pools detected throughout Israel every year since 2000 belonged to the same mosquito species as those identified for USUV (*16*), the geographic discrepancy between circulation of WNV and USUV might be caused by different bird species carrying USUV or WNV. Future studies should examine the circulation of USUV in dead birds, as was demonstrated in several countries in Europe (*11*) and compare bird species carrying USUV with bird species carrying WNV in Israel and those carrying USUV in Europe.

Given the history of flaviviruses that were responsible for major outbreaks worldwide after decades of silent circulation, such as WNV and Zika virus, the accumulating evidence of increased activity of USUV in Europe is alarming. Therefore, investigation of USUV in mosquitoes in Israel is essential, not only because of public health concerns but also because of a need to understand the kinetics and penetration routes of USUV from Africa into Europe, Asia, and the Middle East.
